# Post-graduate Course in Palliative Medicine: Experiences from an E-Learning-Based Pilot Program, a Mixed Methods Study

**DOI:** 10.1177/08258597231171823

**Published:** 2023-05-03

**Authors:** Annamarja Lamminmäki, Minna Hökkä, Outi Hirvonen, Eeva Rahko, Tiina Saarto, Juho T Lehto

**Affiliations:** 1 Department of Clinical Medicine, University of Eastern Finland, Kuopio, Finland; 2 Cancer Center, KYS, 60650 Kuopio University Hospital, Kuopio, Finland; 3 Kajaani University of Applied Sciences, Kajaani, Finland; 4 Faculty of Medicine, University of Oulu, Oulu, Finland; 5 Department of Clinical Oncology, Turku University Hospital, Turku, Finland; 6 Department of Clinical Oncology, University of Turku, Turku, Finland; 7 Department of Oncology, 60664 Oulu University Hospital, Oulu, Finland; 8 Faculty of Medicine, Palliative Care Unit and University of Helsinki, Comprehensive Cancer Center, Helsinki University Hospital, Helsinki, Finland; 9 Faculty of Medicine and Health Technology, 7840 University of Tampere, Tampere, Finland; 10 Department of Oncology, Palliative Care Centre, 60670 Tampere University Hospital, Tampere, Finland

**Keywords:** education, distance, palliative medicine, education, medical, graduate

## Abstract

**Objective:** To study whether E-learning methods are feasible in the post-graduate education of palliative medicine. **Methods:** A mixed-methods study. Evaluations from pilot course attendees were analyzed numerically and answers to open-ended questions about E-learning were analyzed using inductive content analysis. A national pilot E-learning-based post-graduate course in palliative medicine with 24 participating physicians in Finland. The evaluation of teaching modules and different aspects of the course was achieved from the participants through numerical statements and open-ended questions. **Results:** The feedback on most aspects of the course was good. For example, issues of pain and symptom control, lectures, pre-exams, and group discussions were deemed suitable for E-learning, while studying communication and existential issues through E-learning was considered more challenging. The benefits of E-learning included efficacy, better accessibility, and the possibility to go back to the teaching material. Reduced networking and face-to-face interactions were stated as challenges of E-learning. **Conclusions:** E-learning is feasible in the post-graduate education of palliative medicine and can be ‘surprisingly rewarding’. It allows easy access to learn many important topics, while social networking may fall short. Further studies are needed to assess the increase in competence by different learning methods.

## Introduction

The increasing need for palliative care across all levels of healthcare is well recognized.^1,2^ Specialized level palliative care coverage has increased in Europe over the years but is still estimated to be insufficient partly due to the lack of healthcare professionals with a special education in palliative care.^3–5^

The organization of post-graduate, specialist level training in palliative medicine varies between different countries and is a specialty of its own in only a minority of European countries (N. Arias-Casais et al 2019). In Finland, specialist level post-graduate training in palliative medicine leads to a certification of special competence in palliative medicine awarded by the Finnish Medical Association.^6,7^ This education has been available for physicians since 2008 as a national program, including 150 h of theoretical education, a written exam, and work under supervision in a palliative care unit. The majority of the theoretical studies have been offered through a national or Nordic course in palliative medicine including multiple seminar-days with lectures and group discussions.^8,9^ Finland has a population of 5.5 million inhabitants. There are five universities with medical faculties (Helsinki, Turku, Tampere, Oulu, and Kuopio) of which three have a professor in palliative medicine. The long geographical distances and relatively few numbers of national experts pose challenges to the organization of the national curriculum, and these challenges were exacerbated by the travel and meeting restrictions due to the COVID-19 pandemic.

The use of E-learning, meaning educational interventions mediated electronically, usually via the internet,^10^ has increased dramatically during the COVID-19 pandemic.^11^ For post-graduate medical education, novel E-learning-based innovations, and methods have been reviewed for various disciplines including cardiovascular disease,^12^ ophthalmology^13^ and psychiatry.^14^ For palliative care and palliative medicine, E-learning-based educational programs have existed even before the COVID-era at different levels of education and have been shown to be feasible and effective.^15–19^

In Finland, a post-graduate palliative medicine theory course was piloted in 2020-2021 as a part of the EduPal project, a large government financed national project to develop palliative nursing and medical education.^20^ The course was originally designed to integrate distance and contact learning, with emphasis on reflective and interactive learning methods. However, the lockdown due to the global COVID-19 pandemic forced the curriculum to be modified almost entirely to an E-learning format. In this paper, we report the experiences and feedback from this pilot curriculum with an aim to enrich the current knowledge of the usefulness of E-learning methods in the education of palliative medicine.

## Methods

### Participants

Of the total number of 63 applicants, 24 Finnish physicians were selected for the postgraduate course of palliative medicine in October 2020. Selection criteria were a minimum of 2 years working as a physician, the intention to accomplish a special competency in palliative medicine, and former working experience in palliative care. In addition, regional balance was ascertained by selecting four applicants from the Tampere, Turku, Oulu, and Kuopio university hospital areas and eight from the Helsinki University Hospital area, which has over twice as large a population as the other university hospital areas in Finland. Applications were evaluated and selections were made by five authors of this study (JL, TS, AML, ER, OH), who represented the five universities with medical faculties in Finland (see below).

### The Course

The pilot national post-graduate course in palliative medicine was arranged in the EduPal project from December 2020 to December 2021. The curriculum was developed by the professors of palliative medicine of the universities of Tampere and Helsinki (JL and TS), three other physicians with a special competency in palliative medicine representing the universities of Turku, Oulu and Eastern Finland (OH, ER, AML), and the head of the EduPal-project, M. Hökkä, as a representative of palliative nursing to ascertain the multidisciplinary of the course. Other teachers and lecturers in the course were selected from the best national experts on each educational topic. They included physicians with a special competency in palliative medicine and many different background specialties, physicians with a special competency in pain medicine, psychotherapeutics, psychiatrists, psychologists, chaplains, and nurses with a special education in palliative care. In addition, two lectures were provided by international experts online.

The curriculum of the postgraduate course consisted of four modules, each with two seminar days. Details of the curriculum are presented in the Supplemental Material. The topics of the modules were: 1. Pain in palliative care; 2. Communication and psychosocial support, 3. End-of-life care and existential support, and 4. Palliative care in different patient groups. Five mandatory online multiple-choice tests based on the offered prereading material were held before the five seminar days. The seminars concentrated on the advanced aims of the topics, while the basics were assumed to be adopted through previous studies and prereading material. Problem-based learning was facilitated through virtual patient cases, which each participant resolved and sent their answers online before the three seminar days (one for the pain module and two for the different patient groups module). Before three of the seminars, the participants wrote self-reflective essays concerning challenging communication with their own patient, communication and providing psychosocial support in their own working team, and existential challenges in their own patient encounters. Solution proposals for virtual patient cases were evaluated and discussed in the seminars and the self-reflective essays were discussed in small groups of four to six participants arranged in an online platform (Zoom breakout rooms). In addition, expert lectures focusing on each topic at an advanced level were provided in the seminars (see Supplemental material). Each of the 8 seminar days consisted of 8 hours of contact teaching and 4 hours of online prereading, case learning, or educational essays. Thus, a total of 96 h were accepted for the theoretical studies of the special competency of palliative medicine course, which is the current form of specialization for palliative medicine in Finland.^21^

Due to the COVID-19 pandemic, all the teaching and facilitation of learning was performed with E-learning methods online, except for the last seminar on 10 December 2021, which was held as a hybrid session (six participants online and 18 in contact learning). The seven E-learning seminars were arranged on the Zoom platform (Zoom Video Communications, Inc., 2021) and hybrid participation for the last seminar was allowed using the Teams platform (Microsoft 2021).

The prereading material, video-lectures, multiple choice tests, virtual patient cases, and essays were provided through the MoodleTM software of Tampere University (TUNI Moodle). All the seminars were recorded, and both the video and the slides were provided in the Moodle. There was also an interactive discussions area, where both the participants and the experts discussed and shared ideas between the seminar days. The discussion was done asynchronously due to the time restrictions, but answering to the participant’s comments and questions was done as soon as possible by the experts.

### Evaluation of Course

Structured feedback from the participants was achieved after each of the eight seminars through an online questionnaire. A numeric evaluation of different teaching components from four (worst possible) to ten (best possible) included questions on pre-exams, lectures, patient cases, essays, group-based learning, online E-learning, and the seminar as a whole. After the course, a numeric evaluation with the same scale was asked for, which concerned the aspects of the whole curriculum. The grading scale 4-10 was chosen because it is used regularly in Finnish school system. These ratings were treated as continuous data. Written feedback about the modules was collected with open-ended questions and answers concerning the views of the E-learning was evaluated qualitatively.

### Data Analysis

The numeric data of the feedback is presented with numbers, percentages, means, and ranges.

The data of the open-ended questions relating to the overall feedback of each seminar day, the whole curriculum as well as the E-learning provided were analyzed with a qualitative approach namely, inductive content analysis. The manifest content of the data was analyzed. The unit of analysis was either a word, phrase, sentence, or many sentences, which formed a unit of meaning. The analysis followed the three-step process: 1. reduction of the data to reduced expressions; 2. grouping the expressions based on similarities in content; and 3. creating the subcategories and main categories based on their contents.^22^

### Ethical Considerations

Answering the feedback questionnaire was voluntary and done anonymously. All the participants of the course gave their permission to use their answers anonymously in this study. According to Finnish law and research regulations, this type of survey does not need approval from an Ethics Committee.

## Results

The characteristics of the 24 participants of the course are presented in Table 1. Most of the participants had a specialty in general medicine or geriatrics, worked in primary healthcare centers or a hospital at home, and had substantial work experience. The evaluation was retrieved from 23–24 of the 24 participants, depending on the module.

**Table 1.** Characteristics of the Participants (*n*  =  24).

	*n* (%)
Gender	
Female	21 (87)
Male	3 (13)
Time from graduating medical school	
3-5 years	5 (21)
6-10 years	7 (29)
>10 years	12 (50)
Specialty	
General medicine	6 (25)
Geriatrics	5 (21)
Anesthesiology	2 (8)
Internal medicine	1 (4)
Neurology	1 (4)
Pediatrics	1 (4)
Specializing physician^a^	6 (25)
None	2 (8)
Working place	
Secondary or tertiary care hospital	8 (33)
Primary care hospital or hospital at home^b^	16 (67)

^a^ Five specializing in general medicine and one in internal medicine.

^b^ Many of the physicians worked both on the ward and in the hospital at home.

### Numeric Feedback of the Course

The structure of the curriculum with a numeric evaluation for each educational component on a scale from 4 to 10 is presented in Table 2. The feedback was good throughout the curriculum, with high scores, especially for lectures, groupwork, virtual patient cases, and expert discussions (Table 2). The overall mean score for the whole curriculum was 9.54 (range 8–10) and the mean self-reported increase in competence through the course was 9.74 (range 9–10) (Table 3).

**Table 2.** Evaluations of the Educational Component of the Course with Mean Scores and Range for Each on a Scale from 4 (Worst Possible) to 10 (Best Possible). Number of Answers *n*  =  23-24.

	Hours	Evaluation Mean Range	
Module 1: Pain			
Prereading material	*	8.85	7-10
Video lectures	1.5	8.76	4-10
Pre-test	*	8.96	7-10
Lectures Day 1	8	9.00	5-10
3 virtual patient cases	*	9.54	8-10
Pre-assignment	*	9.00	6-10
Lectures Day 2	7	9.01	7-10
Expert discussion of cases Day 2	1	9.46	8-10
Module 2: Psychosocial issues			
Prereading material	*	8.96	7-10
Interactive online material	0.5	8.83	8-10
Pre-test	*	8.38	6-10
Reflective essay	*	8.83	7-10
Lectures Day 1	5	8.98	6-10
Group Workday 1 (essay)	2	9.30	7-10
3 reflective essays	*	8.65	5-10
Video lectures	1.5	9.00	7-10
Lectures Day 2	6	9.24	5-10
Group Workday 2 (essays)	2	9.09	6-10
Module 3: End-of-life			
Prereading material	*	9.21	8-10
Video lectures	1	8.98	7-10
Pre-test	*	8.92	7-10
Lectures Day 1	7	9.17	5-10
Group work Day 1 (PCA)	1	8.88	7-10
Video lectures	1	9.09	7-10
Prereading material	*	8.88	7-10
Reflective essay	*	9.08	7-10
Lectures Day 2	6	9.17	7-10
Group Workday 2 (essays)	2	9.33	7-10
Module 4: Different patient groups			
Prereading material	*	9.25	8-10
Video lectures	1	9.22	6-10
Virtual patient case	*	9.20	6-10
Pre-test	*	8.88	8-10
Lectures Day 1	7	9.43	6-10
Expert discussion of cases Day 1	1	9.25	5-10
Video lectures	0.5	9.22	8-10
Prereading material	*	9.35	8-10
Virtual patient case	*	9.15	6-10
Pre-test	*	8.96	7-10
Lectures Day 2 (hybrid)	6	9.39	6-10
Expert discussion of cases Day 2 (hybrid)	2	9.13	6-10

* Not specified. Total 96 h. See Supplemental material for more details.

**Table 3.** Evaluations of the Curriculum as a Whole and of E-Learning Aspects, with Mean and Range on a Scale from 4 (Worst Possible) to 10 (Best Possible). Number of Answers *n*  =  23-24.

Aspect of curriculum	Mean	Range
Overall	9.52	(8-10)
Usefulness for own work	9.78	(9-10)
Increase in competence	9.74	(9-10)
E-learning, suitability	9.13	(6-10)
E-learning, technical aspects	9.65	(9-10)
Moodle platform, content	9.20	(6-10)
Moodle platform, technical	9.25	(6-10)
Discussions in Moodle, usefulness	8.65	(5-10)
Discussions in Moodle, technical	8.93	(5-10)
Groupwork (online), interactivity	9.21	(6-10)
Groupwork (online), technical	9.14	(6-10)
Interactivity of case discussion, online	8.85	(6-10)
Interactivity of case discussion, hybrid	9.30	(8-10)
Possibility to ask/discuss, online	9.07	(6-10)
Possibility to ask/discuss, hybrid	9.57	(8-10)

The mean score for the *suitability* of teaching through E-learning concerning the feedback of the whole course was 9.13 (range 6–10) with one participant giving a score under 8. This view on E-learning varied between modules with a mean score for psychosocial support and communication of 9.04 (range 5–10), end-of-life care 9.07 (range 7–10), pain 9.27 (range 7–10), and patient groups 9.38 (range 8–10). The overall technical functionality of E-learning was evaluated to be high with a mean score of 9.65 (range 9–10). The technical aspects of the Moodle platform were also rated to be quite good (Table 3). The online groupwork in “breakout rooms” was evaluated to have good interactivity (mean score of 9.21) and a high technical functionality (mean score of 9.14). The possibility to ask and discuss, and the interactivity of case discussions, were rated to be somewhat higher in the one hybrid seminar day than during the online seminar days (Table 3).

### The Physicians’ Views of E-Learning

The content analysis identified four main categories, including a total of 27 subcategories related to the physicians’ views of E-learning in post-graduate palliative medicine education (Table 4).

**Table 4.** Participants Views of E-Learning.

Main category	Subcategory
Methods and issues suitable for E-learning	Lectures are well suited for E-learning Doing exams and sharing materials are easy in E-learning Issues related to pain are suitable for E-learning Issues related to different patient groups and symptom control are suitable for E-learning Group discussions are suitable for E-learning
Wish for future E-learning	Half of the education could be provided by E-learning Most of the education could be provided by E-learning Specialization education should be provided by E-learning COVID-19 has adapted us to learn by E-learning
Benefits of E-learning	E-learning works well The technical connections work well E-learning is surprisingly rewarding Easy to work in groups As good as face-to-face learning Saves the efforts of traveling E-learning made it possible to participate in the course Saves energy for everyday life Easier to concentrate Promotes more efficient learning Possibility le to meet colleagues around the country Possibility to go back to the material
Challenges in E-learning	Networking falls short in E-learning Not all subjects are suitable for E-learning Discussions would be more productive face-to-face E-learning is not as interactive as face-to-face learning It is hard to learn alone at home Technical problems may complicate participation

The main category ‘Methods and issues suitable for E-learning’ included five subcategories (Table 4). In the subcategory ‘*Group discussions are suitable for E-learning’* the participants highlighted the applicability of group discussions in E-learning. In the subcategory ‘*Issues related to pain are suitable for E-learning’* the participants highlighted the suitability of the subject to E-learning. As they expressed:

‘*It positively surprised me. It was even easier, so to say, to open your mouth and ask in discussions.’ [q 6 part 1]*

‘*for pain, E-learning certainly works, because it is more of a “how-to” approach.’* [8]

The main category ‘Wish for future E-learning’ included four subcategories (Table 4). In the subcategory ‘*Covid19 has adapted us to learn by E-learning’* the participants reflected that the pandemic has forced them to learn how to use online tools. They expressed their view that the education could be provided by E-learning in the subcategory ‘*Specialization education should be provided by E-learning’*. Their expressions are given below:

‘*With the Corona pandemic, you get so used to this, that distance learning starts to feel natural, and you learn to use the technology.’* [1]

‘*I encourage you to continue using distance learning!’* [3]

The main category ‘Benefits of E-learning’ included 12 subcategories (Table 4). Some of the participants highlighted that E-learning enabled participation in the subcategory ‘*E-learning made it possible to participate in the course’*. Some of the participants pointed out that E-learning supports learning in the subcategory ‘*Easier to concentrate in E-learning’.* Examples of their expressions are given below*:*

‘*For me, this has been a godsend and enabled me to participate.’* [11]

*‘Personally, I feel that I can concentrate even better remotely.’* [q 6 part 2]

The main category ‘Challenges in E-learning’ included six subcategories (Table 4). In the subcategory ‘*Networking falls short in E-learning’* the participants expressed the lack of networking with colleagues during online education. In addition, they expressed that certain subjects such as communication and existential issues would benefit from the possibility of face-to-face teaching in the subcategory *‘Not all subjects are suitable for E-learning’.* Some examples of their expressions are given below:

‘*Of course, in face-to-face teaching you can have informal discussions during the breaks, which do not take place remotely.’* [18]

‘*The subject area* (communication) *would benefit from face-to-face learning. This is an important topic, and hopefully we can work on it face-to-face in the future!’* [18]

## Discussion

### Main Findings

In this pilot program, we demonstrated that E-learning is feasible for post-graduate training in palliative medicine. Overall evaluations from the participants were excellent with a high self-reported increase in competence and usefulness for own work through the course.

### What This Study Adds

Earlier, E-learning has been shown to be feasible in many healthcare education settings^11,12,14,23–25^ including also some reports in the context of palliative care.^15,16,26^ The self-reported increase in knowledge and skills with short palliative care-related E-learning courses have been reported among nursing assistants^27^ and among rural health professionals.^15^ Also, an increased work satisfaction has been described among general practitioners after one year of interactive training in palliative care.^28^ However, our study enriches the current literature by adding our results of E-learning-based post-graduate course aimed toward special education in palliative medicine.

We found that physicians in training considered E-learning to be feasible in the postgraduate education of palliative medicine. However, as could be expected, the suitability of E-learning varied according to the topic. In our study, there appeared to be differences in the participants’ ratings for suitability between topics and modules, which was also reflected in the qualitative analysis. The suitability for E-learning was rated lowest, though still quite high, for psychosocial and end-of-life issues, and highest for pain and different patient groups. Lectures, exams, the sharing of material, and groupwork were also perceived to be suitable for E-learning. Groupwork in Zoom was performed using the “breakout rooms”^29^ and the interactivity of the groupwork was estimated to be good.

The qualitative analysis revealed a number of benefits of E-learning experienced by the participants.

For some, participation in the program would not have been possible in contact learning, but E-learning made this possible. Related to this was the notion that E-learning saved the effort of traveling. Finland is a rather scarcely populated country with large geographical distances with only a few competent teachers for special-level education in palliative medicine. The organization of the post-graduate level training in palliative medicine is deemed to be most feasible with national collaboration, which however poses challenges with the long geographical distances involved. We believe that the benefits of E-learning that come from minimizing the travel efforts and increasing the possibilities to participate, might be especially salient in other countries with scarce populations and long geographical distances.^15^

Some participants also described that E-learning was more suitable for their learning styles; for example, they experienced that it was easier to concentrate through E-learning. The possibility to review the material also came up as a benefit. In our program, this possibility to review was integral, as the Moodle platform included all the material, including the recorded videos of the lectures, for the whole duration of the program. Earlier discussion about the benefits of E-learning reflect similar issues, with the addition of better cost-effectiveness compared to face-to-face learning,^10,16,30^ but this was not assessed in our study. It is noteworthy that E-learning may have different benefits and challenges for participants with different learning styles.^30^^(p)^

One challenge that the participants experienced was that networking and discussions with colleagues were not as easy as in contact learning. Especially the possibilities for informal networking during the breaks were lost. Still, some of the participants appreciated the possibility to connect with colleagues from far away through the easy access provided by the virtual webinars. This calls for further development of the education in providing informal group discussions or other ways to ensure networking. Finally, it would be important to enable participants to meet each other at an early point in the education to lower the threshold for facilitating discussions and networking through online tools later. The issue of networking may have particular importance in the discipline of palliative medicine, since competence in teamwork, networking, and social interactions can be considered paramount for specialists working in palliative medicine.^27,31^

An adequate technical infrastructure, reliable internet access, and skills in information and communication technology (ICT) are essential for E-learning programs to work.^10,28,32,33^ In our study, the technical aspects and functionality of the platforms were rated as being good. This may not be applicable to all countries, since ICT access and competence are high in Finland.^34,35^ The course that we describe here required ICT competence also from the faculty. The interactive nature of the course required quite a lot of preparations and active participation in the online discussion board between the modules. Thus, facilitating high-quality E-learning requires teaching resources and pedagogical skills, which may occasionally even exceed the competencies needed in face-to-face courses.

#### Limitations

On the larger scales, E-learning has even been reported to be more effective than offline learning in a meta-analysis on knowledge and skills in undergraduate medical education.^36^ However, a Cochrane review including 16 randomized trials about E-learning for different groups of healthcare professionals challenged this through a conclusion statement: “compared to traditional learning, E-learning may make little or no difference in patient outcomes or health professionals’ behaviors, skills or knowledge”.^10,37^ In the present study, we did not measure the actual changes in the competency, skills, or knowledge of the participants, which can be considered to be a major shortcoming of this study. In addition, we used only written patient cases, but virtual standardized patients or simulated patient scenarios should be tested in the future.

## Conclusions

To conclude, physicians find E-learning methods feasible for post-graduate education in palliative medicine. Topics related to symptom control are probably best suitable, but also other aspects such as psychosocial support may be included. Interactive and reflective methods can be used, although face-to-face interaction may be essential for networking and communication learning. We encourage facilitators of palliative medicine education to utilize E-learning methods as a part of their post-graduate curricula and to study the ability of E-learning to increase the competency of physicians specializing in palliative medicine.

## Supplemental Material

sj-docx-1-pal-10.1177_08258597231171823 - Supplemental material for Post-graduate Course in Palliative Medicine: Experiences from an E-Learning-Based Pilot Program, a Mixed Methods Study Supplemental material, sj-docx-1-pal-10.1177_08258597231171823 for Post-graduate Course in Palliative Medicine: Experiences from an E-Learning-Based Pilot Program, a Mixed Methods Study by Annamarja Lamminmäki, Minna Hökkä, Outi Hirvonen, Eeva Rahko, Tiina Saarto and Juho T Lehto in Journal of Palliative Care

## References

[1] Callaway MV Connor SR Foley KM . World health organization public health model: A roadmap for palliative care development. J Pain Symptom Manage. 2018;55(2):S6-S13. doi:10.1016/j.jpainsymman.2017.03.030 28801003

[2] Strengthening of palliative care as a component of integrated treatment throughout the life course Report by the Secretariat. 2014.

[3] Arias-Casais N López-Fidalgo J Garralda E , et al. Trends analysis of specialized palliative care services in 51 countries of the WHO European region in the last 14 years. Palliat Med. 2020;34(8):1044-1056. doi:10.1177/0269216320931341 32519584 PMC7388149

[4] Centeno C Lynch T Garralda E Carrasco JM Guillen-Grima F Clark D . Coverage and development of specialist palliative care services across the World Health Organization European Region (2005-2012): Results from a European Association for Palliative Care Task force survey of 53 countries. Palliat Med. 2016;30(4):351-362. doi:10.1177/0269216315598671 26231421 PMC4800456

[5] Saarto T Finne-Soveri H. Palliatiivisen hoidon ja saattohoidon tila Suomessa : Alueellinen kartoitus ja suositusehdotukset laadun ja saatavuuden parantamiseksi. February 2019. Accessed September 29, 2022. https://julkaisut.valtioneuvosto.fi/handle/10024/161396.

[6] Arias-Casais N Garralda E Rhee JY , et al. EAPC Atlas of palliative care in Europe 2019. 2019. Accessed September 7, 2022. https://dadun.unav.edu/handle/10171/56787.

[7] Finnish Medical Association. https://www.laakariliitto.fi/palvelut/koulutukset/erityispatevyydet/.

[8] Vanhanen A Niemi-Murola L Pöyhiä R . Twelve years of postgraduate palliative medicine training in Finland: How international guidelines are implemented. Palliat Med Rep. 2021;2(1):242-249. doi:10.1089/PMR.2021.0020 34927148 PMC8675218

[9] Sigurdardottir V Edenbrandt CM Hirvonen O Jespersen BA Haugen DF . Nordic specialist course in palliative medicine: Evaluation and impact on the development of palliative medicine in the nordic countries: A survey among participants from seven courses 2003-2017. J Palliat Med. 2021;24(12):1858-1862. doi:10.1089/jpm.2021.0310 34415780

[10] Vaona A Banzi R Kwag KH , et al. E-learning for health professionals. Cochrane Database Syst Rev. 2018;2018(1):49. doi:10.1002/14651858.CD011736.PUB2 PMC6491176 29355907

[11] Ahmady S Kallestrup P Sadoughi M , et al. Distance learning strategies in medical education during COVID-19: A systematic review. J Educ Health Promot. 2021;10(1). doi:10.4103/JEHP.JEHP_318_21 PMC8719547 35071627

[12] Agarwal A Rodrigues Duraes A Omboni S , et al. COVID-19 and the digitalisation of cardiovascular training and education-A review of guiding themes for equitable and effective post-graduate telelearning. Front Cardiovasc Med Wwwfrontiersinorg. 2019;1:666119. doi:10.3389/fcvm.2021.666119 PMC8283504 34277728

[13] Mishra K Boland MV Woreta FA . Incorporating a virtual curriculum into ophthalmology education in the coronavirus disease-2019 era. Curr Opin Ophthalmol. 2020;31(5):380-385. doi:10.1097/ICU.0000000000000681 32694267

[14] Jayaram M Shields G Buisman-Pijlman F . Novel methods of teaching psychiatry to medical and postgraduate students. Curr Opin Psychiatry. 2021;34(5):491-496. doi:10.1097/YCO.0000000000000725 34421112

[15] Koczwara B Francis K Marine F Goldstein D Underhill C Olver I . Reaching further with online education? The development of an effective online program in palliative oncology. J Cancer Educ Off J Am Assoc Cancer Educ. 2010;25(3):317-323. doi:10.1007/S13187-009-0037-6 20119693

[16] Taroco ALC Valente TC de O Carbogim CS . Distance learning for updating health professionals in palliative care: A systematic review. BMJ Support Palliat Care. 2017;7(2):205-211. doi:10.1136/BMJSPCARE-2015-001042 28062410

[17] Pereira J Palacios M Collin T , et al. The impact of a hybrid online and classroom-based course on palliative care competencies of family medicine residents. Palliat Med. 2008;22(8):929-937. doi:10.1177/0269216308094561 18772211

[18] Pereira J Chary S Faulkner J Tompkins B Moat JB . Primary-level palliative care national capacity: Pallium Canada. BMJ Support Palliat Care. 2021:bmjspcare-2021-003036. doi:10.1136/bmjspcare-2021-003036 34315718

[19] Nadeau MC Bilodeau K Daoust L . Using web-based training to optimize pediatric palliative care knowledge transfer. Can Oncol Nurs J Rev Can Nurs Oncol. 2020;30(1):31-37. doi:10.5737/236880763013137 PMC7585705 33118985

[20] Basic information of the project - Palliatiivisen koulutuksen kehittäminen. Accessed September 7, 2022. https://www.palliatiivisenkoulutuksenkehittaminen.fi/basic-information-of-the-project/.

[21] Lääkäriliitto - Palliatiivinen lääketiede. Accessed September 7, 2022. https://www.laakariliitto.fi/palvelut/koulutukset/erityispatevyydet/palliatiivinen/.

[22] Elo S Kyngäs H . The qualitative content analysis process. J Adv Nurs. 2008;62(1):107-115. doi:10.1111/J.1365-2648.2007.04569.X 18352969

[23] Camargo CP Tempski PZ Busnardo FF de Arruda Martins M Gemperli R . Online learning and COVID-19: A meta-synthesis analysis. Clin Sao Paulo Braz. 2020:75. doi:10.6061/CLINICS/2020/E2286 PMC7605278 33174948

[24] Gupta A Haritha D Ramachandran R , et al. Nationwide impact of COVID-19 pandemic on postgraduate medical teaching, research, and mental stress: A cross-sectional online questionnaire-based survey. J Anaesthesiol Clin Pharmacol. 2022;38(Suppl 1):S34-S45. doi:10.4103/joacp.JOACP_673_20 PMC9438838 36060181

[25] Zhou T Huang S Cheng J Xiao Y . The distance teaching practice of combined mode of massive open online course micro-video for interns in emergency department during the COVID-19 epidemic period. Telemed e-Health. 26(5):584-588. doi:10.1089/tmj.2020.0079 32271650

[26] Pulsford D Jackson G Yates S O’brien T Duxbury J . Classroom-based and distance learning education and training courses in end-of-life care for health and social care staff: A systematic review. Palliat Med. 2013;27(3):221-235. doi:10.1177/0269216311429496 22126845

[27] Ersek M Wood BB . Development and evaluation of a nursing assistant computerized education programme. Int J Palliat Nurs. 2008;14(10):502-509. doi:10.12968/IJPN.2008.14.10.31495 18978697

[28] General practitioners’ attitudes and ethical decisions in end-of-life care after a year of interactive Internet-based training - ProQuest. Accessed September 25, 2022. https://www.proquest.com/docview/221661342?accountid=11739. 10.1080/08858190209528785 12000099

[29] Rucker J Steele S Zumwalt J Bray N . Utilizing zoom breakout rooms to expose preclerkship medical students to TeleMedicine encounters. Med Sci Educ. 2020;30(4):1359-1360. doi:10.1007/S40670-020-01113-W 33078081 PMC7556762

[30] (PDF) E-learning: A review of literature. Accessed September 25, 2022. https://www.researchgate.net/publication/331938876_E-learning_A_review_of_literature.

[31] Melender HL Hökkä M Saarto T Lehto JT . The required competencies of physicians within palliative care from the perspectives of multi-professional expert groups: A qualitative study. BMC Palliat Care. 2020;19(1):65. doi:10.1186/s12904-020-00566-5 32386513 PMC7211329

[32] Katlen JN Manlapaz MR Hoffman A . Considerations for appropriateness of virtual learning in the postpandemic environment. J Nurs Educ. 2022;61(9):503-509. doi:10.3928/01484834-20220705-04 36098542

[33] Vermisli S Cevik E Cevik C . The effect of perceived stress and digital literacy on student satisfaction with distance education. Rev Esc Enferm USP. 2022;56:e20210488. doi:10.1590/1980-220X-REEUSP-2021-0488EN PMC10116882 36082985

[34] Aleksejeva V Lavrinenko O Betlej A Danileviča A . Analysis of disparities in the use of information and communication technology (ICT) in the EU countries. Entrepreneurship Sustain Issues. 2021;9(2):332-345. doi:10.9770/jesi.2021.9.2(22)

[35] Tilastokeskus - Tilastot aiheittain - Väestön tieto- ja viestintätekniikan käyttö. Accessed September 6, 2022. https://www.tilastokeskus.fi/til/sutivi/index.html.

[36] Pei L Wu H . Does online learning work better than offline learning in undergraduate medical education? A systematic review and meta-analysis. Med Educ Online. 2019;24(1). doi:10.1080/10872981.2019.1666538 PMC6758693 31526248

[37] Swift A. E-learning may be no better than traditional teaching for continuing education of health professionals. Evid Based Nurs. 2019;22(2):52. doi:10.1136/ebnurs-2018-102927 30504452

