# Supplementary material for: Post-graduate Course in Palliative Medicine: Experiences from an E-Learning-Based Pilot Program, a Mixed Methods Study
Source: J Palliat Care. 2023 May 3;39(3):209–16. doi: 10.1177/08258597231171823 (PMC11097605; doi:10.1177/08258597231171823)
Supplement: sj-docx-1-pal-10.1177_08258597231171823 - Supplemental material for Post-graduate Course in Palliative Medicine: Experiences from an E-Learning-Based Pilot Program, a Mixed Methods Study [file sj-docx-1-pal-10.1177_08258597231171823.docx]

Supplementary material

**Curriculum of the post-graduate course in palliative medicine**

**MODULE 1: Pain in Palliative Care**

Prereading material before the first seminar day

- Pain. Current care guidelines. Working group set up by the Finnish Medical Society Duodecim, the Finnish Society of Anesthesiology and the Finnish Society of General Medicine 2015. Available online: [www.kaypahoito.fi](http://www.kaypahoito.fi)
- Palliative Care and End-of-life Care. Current care guidelines. Working group set up by the Finnish Medical Society Duodecim and the Finnish Society of Palliative Medicine 2019. Available online: [www.kaypahoito.fi](http://www.kaypahoito.fi)
- Kalso E, Haanpää M, Hamunen K, Konttinen V, Vainio A. Pain. Finnish Medical Society Duodecim. 4th ed. Helsinki 2018. pp. 56-85 (Physiology and mechanisms of pain), pp. 108-118 (Acute and chronic pain), pp. 138-144 (Clinical examination of a patient with pain), pp. 144-160 (Neurophysiological tests in the evaluation of a patient with pain), pp. 161-165 (Basics of Psychological evaluation), pp. 177-221 (Pharmacologic management of pain), pp. 222-230 (Local anesthesia and neuroablative techniques), pp.242-252 (Physiotherapeutic methods), pp. 253-259 (Psychological methods), pp. 330-344 (Neuropathic pain), pp. 535-543 (Pain in elderly), pp. 546-577 (Cancer pain).

Topics of the three video-lectures before the first seminar day:

- Mechanisms and background of pain
- Clinical examination of a patient with pain
- Basics of pain management

Pre-test before the first seminar day

- Online multiple-choice test with 15 questions based on the prereading material and video-lectures

*First E-learning seminar 4 December 2020*

- Topics of the lectures:
  - Pharmacology and use of opioids in palliative care
  - NSAIDs and paracetamol
  - Management of neuropathic pain
  - Patients with renal or liver insufficiency
  - Elderly patients
  - Pain refractory to pain medication
  - Practical challenges in pain medication – prescribing, evaluating the efficacy, storing and disposing pain medication
  - Points of view by a psychologist in pain management
  - Non-pharmacological pain management in the context of palliative care
  - Invasive techniques in pain management
- Interactive discussions after the lectures (1 hour altogether)

Three virtual patient cases before the second seminar day:

- All the participants wrote essay responses on how they would have managed the three virtual patient cases concerning pain management in the context of palliative care
- The participants returned their answers online through the Moodle platform
- The teachers of the course evaluated each answer for the feedback session in the seminar Day 2 based on these answers. In addition, the participants were able to see each other’s answers to the cases and discuss them in the Moodle platform before the seminar.

*Second E-learning seminar 22 January 2021*

- Interactive discussion and expert presentations of the three virtual cases (2.5 hours)
- Interactive discussion about the issues brought up in the Moodle discussion area (1 hour)
- Topics of the lectures:
  - Non-malignant pain in palliative care
  - Cannabinoids
  - Ketamine
  - When to consult a specialist in pain management?
  - Oral Pain
  - Pain in a patient with a cardiological or respiratory disease
  - Patient with drug abuse

**MODULE 2: Communication and Psychosocial support**

Prereading material before the first seminar day

- Hietanen P, Kaleva-Kerola J, Pyörälä P. Communication between a patient and a physician. Finnish Medical Society Duodecim. 1st ed. Helsinki 2020. pp. 14-38 (Basics of good communication), pp. 87-94 (Discussing with the closest ones), pp. 143-150 (Discussing difficult issues), pp. 151-158 (Discussing palliative care), pp. 159-162 (Advance care planning together with the patient), pp. 163-168 (Discussing the Do Not Resuscitate -order), pp. 169-180 (Discussing with the patient and their closest ones when facing death), pp. 191-196 (Complying with the illness), pp. 197-204 (Emotions related to an illness and communication), pp. 205-211 (Maintaining hope).
- Laaksovirta H. Discussing a serious illness with a patient and family. Duodecim 2017;133:1076-80.

Online course: “Communication with a patient with a serious disease”. Finnish Medical Society Duodecim 2019. <https://www.oppiportti.fi/op/dvk00139>. (About 1 hour E-learning course).

Pre-test before the first seminar day.

- Online multiple-choice test with 10 questions based on the prereading material and online course.

Reflective essay before the first seminar day (1-2 pages).

- Own patient case with challenging communication and description of own strengths and weaknesses in communication
- The participants returned their essays online through the Moodle platform
- The teachers of the course evaluated each answer and planned the interactive group sessions for the first seminar day based on them. In addition, the participants were able to see each other’s essays and discuss them in the Moodle platform before the first seminar day.

*First E-learning seminar 19 March 2021*

- Interactive discussions about the reflective essays and communication. Small groups of 4-5 participants and an expert facilitator in Zoom breakout rooms (2 hours).
- Topics of the lectures:
  - Psychosocial support and the physician’s work
  - Development of good practices for supportive discussions in the care of patients with incurable diseases
  - Different models of psychotherapy in palliative care
  - Communication: Why and how?
  - Communication: What have we learned? (Summary of the day)

Three reflective essays before the second seminar day (1-2 pages each).

- Essays:
  1. Going back to my own case from the first seminar day (Would I have done something differently based on what I have learned? How could I improve my communication in general?)
  2. What is good and what should be developed in my own working team regarding communication and psychosocial support?
  3. How do I take care of my own coping?
- The participants returned their essays online in the Moodle platform
- The teachers of the course evaluated each answer and planned the interactive group sessions for the second seminar day based on them. In addition, the participants were able to see each other’s essays and discuss them in the Moodle platform before the second seminar day.

Topics of the four video-lectures before the second seminar day:

- Taking children into account during the serious illness of a parent
- Encountering the patient
- Emotional skills and validation
- Coping of a professional in palliative care

*Second E-learning seminar 23 April 2021*

- Interactive discussions about the reflective essays and communication. Small groups of 4-5 participants and an expert facilitator in Zoom breakout rooms (2 hours).
- Topics of the lectures:
  - Supporting the family and children
  - Conflicts with the closest ones
  - Mindfulness and awareness skills
  - Coping of the helper
  - Basics of dignity therapy

**MODULE 3: End-of-life care and existential support**

Prereading material before the first seminar day:

- Saarto T, Hänninen J, Antikainen R, Vainio A. Palliative care. Finnish Medical Society Duodecim. 3rd ed. Helsinki 2015. pp. 255-278 (End-of-life care).
- Saarto T, Finne-Soveri H and expert working groups. State of palliative and terminal care in Finland. Regional survey and proposals to improve the quality and availability of care. Finnish Ministry of Social Affairs and Health 2019:14. <http://urn.fi/URN:ISBN:978-952-00-4041-3>
- Saarto T, Finne-Soveri H and expert working groups. Recommendation on the provision and improvement of palliative care services in Finland. Finnish Ministry of Social Affairs and Health 2019:68. <http://urn.fi/URN:ISBN:978-952-00-4126-7>
- Tasmuth T. End-of-life of a patient with a brain tumor. Duodecim 2020;136:1233-41.
- Kiljunen M, Marjamäki E. Evaluating drug therapy in end-of-life care. Finnish Medical Journal 2017; 19:1231-1235.
- Voluntary reading not included in the pre-test: Cherny N et al. (eds.). Oxford Textbook of Palliative Medicine. Oxford University Press 2015. pp. 1025-1141 (Terminal Phase).

Topics of the three video-lectures before the first seminar day:

- Advance care planning and setting goals of care
- General symptoms (Fatigue, Cachexia, Anorexia)
- Patient controlled analgesia device

Pre-test before the first seminar day

- Online multiple-choice test with 12 questions based on the prereading material and video-lectures

*First E-learning seminar 17 September 2021*

- Working groups in Zoom breakout rooms concerning patient cases of drug infusions in end-of-life care (1 hour).
- Topics of the lectures:
  - Patient controlled analgesia device and drug infusions in end-of-life care
  - Palliative sedation: How, when, and to whom?
  - Palliative sedation: Practical issues with patient cases
  - End-of-life care at home
  - End-of-life care at a nursing home
  - End-of-life care in the emergency room
  - Practical and juridical issues related to the death of a patient
  - Cachexia, fatigue, and parenteral hydration with patient cases
  - Death in different cultures

Reflective essay before the second seminar day (1-2 pages).

- Own patient case with challenging existential questions (Patient’s fear of death or difficulty to cope with the disease. What felt difficult or challenging when caring for the patient?)
- The participants returned their essays online through the Moodle platform
- The teachers of the course evaluated each answer and planned the interactive group sessions for the second seminar day based on them. In addition, the participants were able to see each other’s essays and discuss them in the Moodle platform before the second seminar day.

Topics of the two video-lectures before the second seminar day:

- Total pain
- Existential suffering

Voluntary prereading material before the second seminar day (no pre-test)

- Cherny N et al (eds.). Oxford Textbook of Palliative Medicine. Oxford University Press 2015. pp. 1059-1122.
- Best M, Leget C, Goodhead A, Paal P. An EAPC white paper on multi-disciplinary education for spiritual care in palliative care. BMC Palliative Care 2020; 19: 9.
- Haho A. Existential suffering in cancer patients in the palliative care phase. Finnish Medical Journal 2017;33:1704-1709.
- Sipola V, Pöyhiä R, Anttonen MS, Pajunen M. Supporting patients’ spirituality and existential issues in palliative care. National recommendation. Evangelical Lutheran Church of Finland 2021. ISBN 978-951-789-715-0, ISSN 2341-9407. [Kirkon julkaisut (evl.fi)](https://julkaisut.evl.fi/catalog/Tutkimukset%20ja%20julkaisut/r/4282/viewmode=infoview).

*Second E-learning seminar 15 October 2021*

- Expert panel discussion: Encountering existential suffering in challenging situations. (1.5 hours)
- Interactive discussions about the reflective essays on existential suffering. Small groups of 4 participants and an expert facilitator in Zoom breakout rooms (1.5 hours).
- Topics of the lectures:
  - Developing spiritual care and EAPC recommendations
  - Existential pain among Finnish patients
  - Measurement tools of spirituality
  - Recommendations of spiritual care for Finnish care practices
  - Permission for grief and life – How to support the recovery of the closest one?
  - Guilt as a part of the process of grief
  - Existential pain in art

**MODULE 4: Palliative care in different patient groups**

Prereading material before the first seminar day

- Saarto T, Hänninen J, Antikainen R, Vainio A. Palliative care. Finnish Medical Society Duodecim. 3rd ed. Helsinki 2015. pp. 113-154 (Gastrointestinal symptoms), pp. 359-418 (Palliative care in cancer), pp 448-453 (Spinal compression), pp. 458-463 (Malignant bowel obstruction), pp. 493-510 (Cardiovascular diseases in end-of-life).
- Hill L, Geller TP, Baruah R et al. Integration of a palliative approach into heart failure care: a European Society of Cardiology Heart Failure Association position paper. Eur J Heart Fail 2020;22:2327-2339.

Topics of the four video-lectures before the second seminar day:

- Palliative care of a patient with heart failure
- Palliative care of a patient with liver cirrhosis
- Nausea
- Skin in palliative care

Pre-test before the first seminar day

- Online multiple-choice test with 12 questions based on the prereading material and video-lectures

Virtual patient cases before the first seminar day

- All the participants wrote essay responses on how they would manage the virtual patient case. Half of the participants responded to a virtual case of a heart failure patient and the other half to a virtual case of a malignant bowel obstruction.
- The participants returned their answers online through the Moodle platform
- The teachers of the course evaluated each answer and formed the feedback session in the seminar based on these answers. In addition, the participants were able to see each other’s answers to the cases and discuss them in the Moodle platform before the seminar day.

*First E-learning seminar 12 November 2021*

- Interactive discussion and expert presentations of the two virtual cases:
  - Patient with heart failure (1 hour)
  - Patient with malignant bowel obstruction (1 hour)
- Topics of the lectures:
  - Diuretics and other heart medications
  - Risk of thrombosis and bleeding
  - End-of-life care pathway for a patient with heart disease
  - Surgical management of a malignant bowel obstruction
  - Conservative management of a malignant bowel obstruction
  - Ascites
  - Local symptoms in head and neck cancer
  - Local symptoms in gynecological cancers
  - Palliative radiotherapy

Prereading material before the second seminar day:

- Saarto T, Hänninen J, Antikainen R, Vainio A. Palliative care. Finnish Medical Society Duodecim. 3rd ed. Helsinki 2015. pp. 91-111 (Respiratory symptoms), pp. 465-475 (Chronic lung diseases), pp. 511-536 (Neurological diseases), pp. 551-570 (Palliative care of the elderly).
- Andersen PM, Abrahams S, Borasio GD et al. EFNS guidelines on the Clinical Management of Amyotrophic Lateral Sclerosis (MALS) – revised report of an EFNS task force. Eur J Neurol 2012; 19: 360–375.
- Antikainen R, Konttila T, Virolainen J, Strandberg T. End-of-life care of a patient with advanced dementia. Finnish Medical Journal 2013;22:909 – 915.

Topics of the two video-lectures before the second seminar day:

- Palliative care of a patient with chronic obstructive pulmonary disease
- About palliative care of a patient with amyotrophic lateral sclerosis

Pre-test before the second seminar day

- Online multiple-choice test with 12 questions based on the prereading material and video-lectures

Virtual patient cases before the first seminar day

- The participant who was a pediatrician made a presentation of her own pediatric patient case, which was evaluated and commented in the seminar by a teacher who was a pediatrician and had a special competency in palliative medicine.
- All the other participants wrote essay responses on how they would manage the virtual patient case. Half of these participants responded to a virtual case of a patient with advanced pulmonary disease and the other half to a virtual case of a patient with advanced dementia.
- The participants returned their answers online through the Moodle platform
- The teachers of the course evaluated each answer and formed the feedback session in the seminar based on these answers. In addition, the participants were able to see each other’s answers to the cases and discuss them in the Moodle platform before the seminar day.

*Second seminar day 10 December 2021 (Hybrid teaching)*

- Interactive discussion and expert presentations of the two virtual cases
  - Patient with advanced pulmonary disease (1 hour)
  - Patient with advanced dementia (1 hour).
- Presentation of a pediatric case to the course including discussion (30 min)
- Topics of the lectures:
  - Opioids in the management of respiratory symptoms
  - Non-invasive ventilation and other similar therapies
  - Oxygen and airflow
  - Palliative care for ALS patients in the hospital district of Helsinki and Uusimaa
  - End-of-life care in patients with ALS
  - Goals and limits of care in elderly patients with multimorbidity or dementia
  - Delirium in a patient with dementia
- Special program for the pediatrician during the Interactive discussion and lectures concerning dementia (developing palliative care for children in Finland).
